# High photon-to-heat conversion efficiency in the wavelength region of 250–1200 nm based on a thermoelectric Bi_2_Te_3_ film structure

**DOI:** 10.1038/srep44614

**Published:** 2017-03-16

**Authors:** Er-Tao Hu, Yuan Yao, Kai-Yan Zang, Xin-Xing Liu, An-Qing Jiang, Jia-Jin Zheng, Ke-Han Yu, Wei Wei, Yu-Xiang Zheng, Rong-Jun Zhang, Song-You Wang, Hai-Bin Zhao, Osamu Yoshie, Young-Pak Lee, Cai-Zhuang Wang, David W. Lynch, Jun-Peng Guo, Liang-Yao Chen

**Affiliations:** 1 School of Optoelectronic Engineering, Nanjing University of Posts and Telecommunications, Nanjing, China; 2 Department of Optical Science and Engineering, Fudan University, Shanghai, China; 3 Graduate School of IPS, Waseda University, Fukuoka, Japan; 4 Department of Physics, Hanyang University, Seoul, Korea; 5 Department of Physics, Iowa State University, Ames, Iowa, USA; 6 Department of Electrical & Computer Engineering, University of Alabama in Huntsville, Alabama, USA

## Abstract

In this work, 4-layered SiO_2_/Bi_2_Te_3_/SiO_2_/Cu film structures were designed and fabricated and the optical properties investigated in the wavelength region of 250–1200 nm for their promising applications for direct solar-thermal-electric conversion. A typical 4-layered film sample with the structure SiO_2_ (66.6 nm)/Bi_2_Te_3_ (7.0 nm)/SiO_2_ (67.0 nm)/Cu (>100.0 nm) was deposited on a Si or K9-glass substrate by magnetron sputtering. The experimental results agree well with the simulated ones showing an average optical absorption of 96.5%, except in the shorter wavelength region, 250–500 nm, which demonstrates the superior absorption property of the 4-layered film due to the randomly rough surface of the Cu layer resulting from the higher deposition power. The high reflectance of the film structure in the long wavelength region of 2–20 μm will result in a low thermal emittance, 0.064 at 600 K. The simpler 4-layered structure with the thermoelectric Bi_2_Te_3_ used as the absorption layer may provide a straightforward way to obtain solar-thermal-electric conversion more efficiently through future study.

At present, there are mainly two well-established methods of capturing solar energy for human benefit: (1) photo-to-electron and (2) photo-to-thermal conversion^1^,^2^. Photovoltaic cells are based on the principle of converting the photons into electricity by a photon absorption process in which the electron-hole pairs are generated. In the photo-thermal processes, heat can be generated by the device by efficiently absorbing the incident photons from the solar radiation with applications such as solar-heating, solar-thermal-electricity generation, solar-thermoelectrics, solar-thermophotovoltaics, and so on^1^,^3^,^4^,^5^,^6^.

For solar-thermoelectrics, the heat accumulated in the devices can be converted directly into electricity by properly using some kind of thermoelectric material. To date, a peak efficiency of 7.4% for a concentrating solar thermoelectric device has been achieved^1^,^4^, but it is lower than the theoretically predicted efficiency of 24%^4^ and is also lower than that of conventional photovoltaic cells. By an effort to improve the efficiency, it will be possible for a solar thermoelectric device to have an efficiency matching that of many other solar power devices in applications^7^,^8^.

In solar-to-thermal conversion processes, selective solar absorbers will play a significant role of efficiently absorbing most of the solar energy in the region of radiated wavelengths while suppressing the infrared re-radiation induced by the heating of the selective absorber^3^,^5^,^9^. Six typical structures of selective solar absorbers have been proposed including (a) intrinsic absorbers, (b) semiconductor–metal tandems, (c) multilayer absorbers, (d) cermets, (e) textured absorbers, and (f) photonic crystals^3^,^10^. Among them, multilayered thin-film structures consisting of alternating metal and dielectric layers show the unique advantage of excellent spectral properties in both of broad solar spectral and wide incident angle regions, low thermal emittance, and good thermal stability, making them particularly suitable for high efficiency photo-to-thermal conversion under medium and high temperature conditions^11^,^12^,^13^,^14^,^15^,^16^. In a typical dielectric/metal/dielectric/metal multilayered film structure, incident solar radiation in a wide wavelength range can be absorbed by the partially transparent metal layer due to the multiple reflections at the interface between layers in the multilayered film structure^3^.

In this work, we studied and designed a new type of multilayer structure in which, instead of using transition metals, a thermoelectric material (Bi_2_Te_3_) was used as the key absorption layer in the 4-layered structure SiO_2_/Bi_2_Te_3_/SiO_2_/Cu. By optimally matching the optical amplitude and phase of the light interacting with the structure, a high and average absorptance of 96.5% in the wavelength region of 250–1200 nm is achieved.

## Results

### Numerical simulations

**Figure 1:**
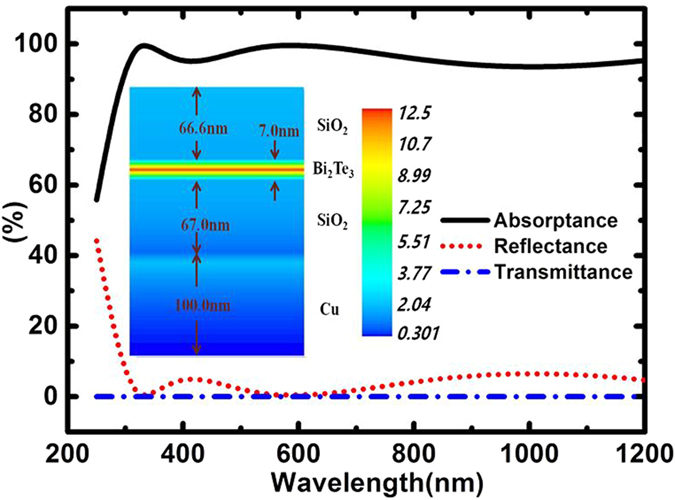
Simulated spectra for absorptance, transmittance and reflectance of the 4-layered film structure for normal incidence. The spectrum of absorptance (solid line) is calculated with the optical constants obtained from the thin Bi_2_Te_3_ film. The inset shows the electric field distribution (λ = 500 nm) of the 4-layered film structure.

A high absorptance of about 95.0% in the wavelength region of 250–1200 nm is achieved for the following film structure parameters used in the simulation: SiO_2_ (66.6 nm)/Bi_2_Te_3_ (7.0 nm)/SiO_2_ (67.0 nm)/Cu (>100.0 nm) with the spectral results shown in Fig. 1. By using the plane-wave-based transfer matrix method^17^,^18^, the electric field distribution was calculated with the result (*λ* = 500 nm) shown in the inset of Fig. 1. It can be seen that the electric field is concentrated mainly in the Bi_2_Te_3_ layer.

### Spectral measurement

**Figure 2:**
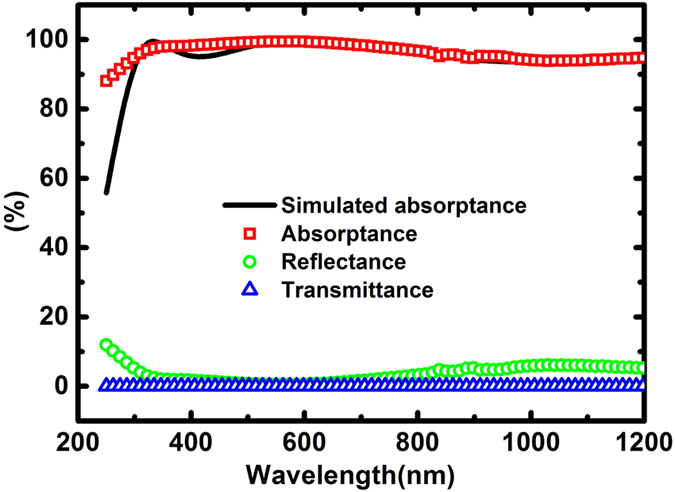
Measured spectra of the absorptance, transmittance and reflectance under the near normal-incidence condition for the samples in the wavelength region of 250–1200 nm as compared with the simulated one.

The experimental results for the optical absorptance, reflectance and transmittance spectra of the samples, with a comparison to the simulated absorptance spectrum, are shown in Fig. 2. A high and average absorptance of about 96.50% was obtained in the wavelength region of 250–1200 nm. It can be seen that experimental results agree very well with the simulated one in the wavelength region of 500–1200 nm. However, in the wavelength region of 250–500 nm, there is some discrepancy, especially in the ultraviolet spectral region where the experimental value of absorptance is even higher than that of the simulated one.

For the measured spectra shown in Fig. 2, the sample was fabricated under the conditions in which the deposition power for the SiO_2_ and Cu layers was 100 W with an Argon gas flow rate of 70 SCCM (standard cubic centimeters per minute), and for the Bi_2_Te_3_ layer was 30 W with an Argon flow rate of 60 SCCM.

**Figure 3:**
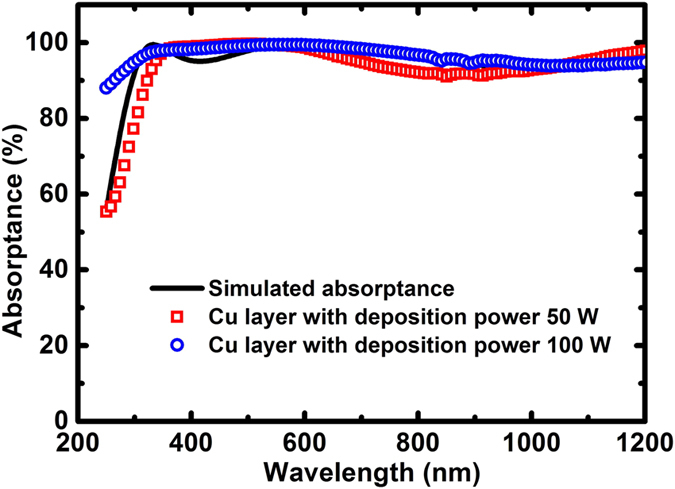
Measured absorptance spectra of the samples under the near normal-incidence condition with different deposition power for the Cu layers (the solid line represents the simulated absorptance spectra).

With the same deposition conditions for SiO_2_ and Bi_2_Te_3_, but by changing the deposition power which is fixed at 50 W for the Cu layer, the measured absorptance spectum for the new sample is shown in Fig. 3, with a comparison to that of the sample with the Cu layer deposited at 100 W of power.

**Figure 4:**
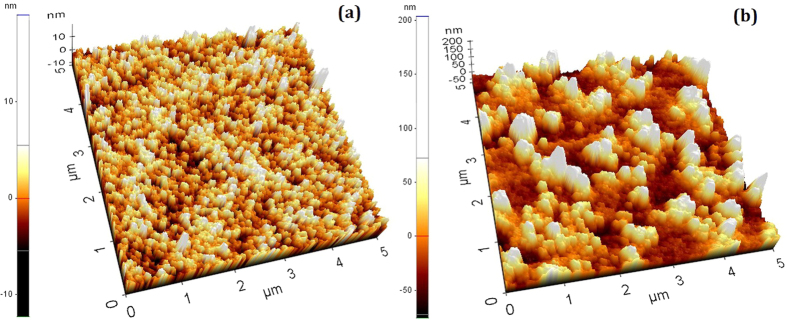
AFM images for the Cu layers deposited under different power conditions (**a**) 50 W, (**b**) 100 W.

**Figure 5:**
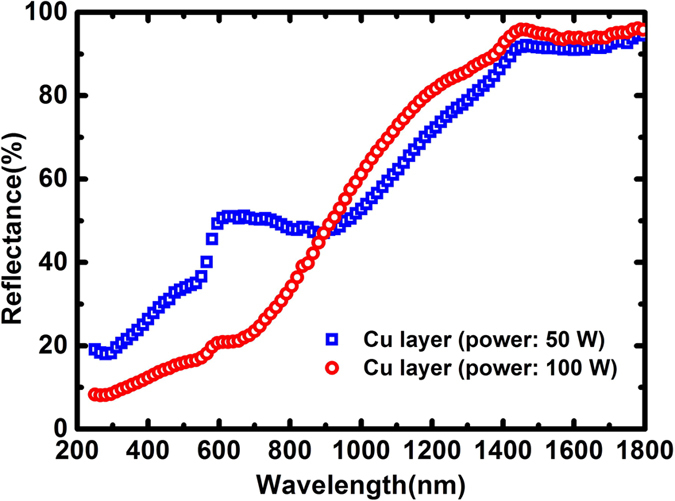
Measured reflectance spectra for the Cu layers deposited under different power.

To investigate the surface properties of the Cu layer under different deposition power conditions, the surface roughness and reflectance spectra were measured by AFM and an integrating sphere spectrophotometer with the results shown in Figs 4 and 5, respectively. It can be seen in Fig. 5 that the reflectance of the Cu layer in the visible wavelength region with a deposition power of 100 W is lower than that of the Cu layer deposited 50 W of power. This may explain why the surface of the Cu layer with a deposited power of 100 W looks blacker than the one deposted at 50 W power.

The surface roughness of the Cu layer is about 2.8 nm and 37.1 nm at the deposition powers of 50 W and 100 W, respectively. Thus, this may reasonably explain that the enhancement of the absorption in the ultraviolet region can be attributed to the light trapping effect of the random rough surface of the Cu layer deposited under the higher-power condition. The measured surface roughness of the 4-layered structures is about 3.9 nm and 70.4 nm for Cu layer deposition at the power of 50 W and 100 W, respectively. The relatively higher surface roughness of 70.4 nm of the 4-layered film structure for the Cu reflection layer deposited at the power of 100 W can well explain the spectral difference apparently showing in the shorter wavelength region of 250–500 nm where the ratio of surface roughness to the wavelength is higher than that in the long wavelength region.

**Figure 6:**
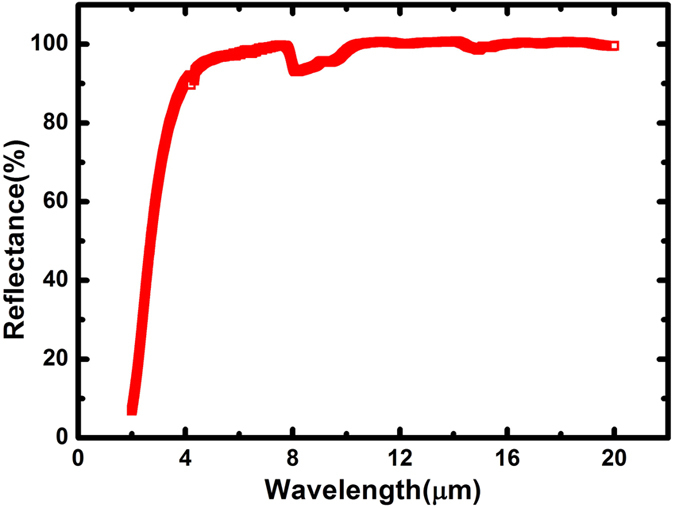
The measured reflectance spectrum of the 4-layered film sample in the 2–20 μm wavelength region.

The reflectance spectrum in the 2–20 μm wavelength region was measured at near normal incidence with the result shown in Fig. 6. The reflectance increases with wavelength and tends to be close to 100% in the longer wavelength region.

The thermal emittance *ε (θ, T*) is a function of incident angle *θ* and temperature *T* and thus can be evaluated with the reflectance spectrum *R( θ, λ*) and Plank’s black body radiation spectrum *E( T, λ*)^19^ as:


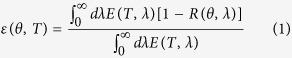


where


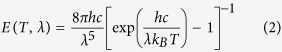


The reduced emittance *ε* (0, *T*) is shown in Table 1. We suggest that the most optimal condition for Bi_2_Te_3_ is to work at a temperature of about 400 K^20^. The highest figure-of-merit with a thermal emittance as low as 0.023 can be achieved, indicating very low thermal radiation corresponding to low energy loss from the sample.

From the detailed analysis shown above, the SiO_2_/Bi_2_Te_3_/SiO_2_/Cu film structure has good spectral properties with the incident light mainly absorbed by the thermoelectric Bi_2_Te_3_ layer. In terms of the heat-to-electron conversion in the next step, therefore, it is possible for the electricity or other information arising from the photon-to-electron conversion process to be extracted in a more straightforward way from the simpler film structure provided with further studies in the future.

## Discussion

In summary, the thermoelectric material Bi_2_Te_3_ was used as the photon absorption layer in the 4-layered SiO_2_/Bi_2_Te_3_/SiO_2_/Cu selective solar absorbers with a high and average absorptance (96.50%) achieved in the wavelength region of 250–1200 nm. The experimental results are in good agreement with the simulated ones except in the short wavelength region of 250–500 nm where the absorber shows superior photon-to-heat conversion property than the simulated ones. In terms of data analysis, the phenomena can be explained due to the random rougher surface of the thicker Cu layer deposited at higher power. The high reflectance of the 4-layered film sample in the longer wavelength region leads to a low thermal emittance to efficiently suppress the thermal radiation. Therefore, good photo-thermal conversion properties have been demonstrated for the samples. In order to extract electricity in a straightforward way from this simpler film structure, more research will be required with the promising and practical applications for the direct solar-thermal-electric conversion in the future.

## Methods

### Optical simulations

The ideal selective solar absorbers will have the characteristic of absorbing solar radiation in the shorter wavelength region up to a certain cutoff wavelength while reflecting light perfectly in the region above the cutoff-wavelength, like a step function^3^. In terms of the law of energy conservation *T* + *R* + *A* = 1, where *T, R* and *A* represent the transmittance, reflectance and absorptance of the light interacting with the material and structure, respectively. This will imply that for the idea situation, selective solar absorbers should have features *T* = 0, *R* = 0 and *A* = 1 in the main solar spectrum region. For metal/dielectric-based solar selective absorbers, the thick metal layer (>100 nm) at the bottom not only acts as a good infrared reflector to reflect the light in the long wavelength region, but also makes the transmittance of the film structure negligible (*T* ≈ 0). In data simulations, the spectral properties of the 4-layered structure (SiO_2_/Bi_2_Te_3_/SiO_2_/Cu) on Si substrate were numerically calculated based on the transfer matrix method. The optical constants of SiO_2_, Si and Cu were obtained from the literature^21^, while for Bi_2_Te_3_, its optical constants were measured by a spectroscopic ellipsometer^22^,^23^ on the thin Bi_2_Te_3_ film deposited by DC (direct current) magnetron sputtering. The electric field distribution was calculated by using the plane-wave-based transfer matrix method^17^,^18^.

### Fabrication

The Cu, Bi_2_Te_3_ and SiO_2_ with a target purity of 99.99% were deposited by DC and RF (radio frequency) magnetron sputtering on Si or K9 glass substrates in a Leybold LAB600SP chamber at room temperature with a background pressure of 6.0 × 10^−6^ mbar. For deposition of the SiO_2_ and Cu layers, the power was 100 W and the Argon gas flow rate was 70 SCCM. The Bi_2_Te_3_ layer was deposited at 30 W with Argon flow rate at 60 SCCM. The film growth rate for each layer was calibrated in advance by deposition time with fixed power and Argon gas flow rate. For the Cu layer, the sputtering rate was calibrated by using a step profiler, while for SiO_2_, a spectroscopic ellipsometer was used. The Bi_2_Te_3_ is the vital layer with its growth rate determined by fitting the measured transmittance spectra in 250–1200 nm wavelength region.

### Optical and surface morphology measurements

Due to the thicker Cu layer (>100 nm) used in the 4-layered film structure, the light transmission through the samples can be ignored, implying *T* ≈ 0. Then the absorptance can be reliably determined from the measured reflectance spectra by *A* ≈ 1-*R* based on the law of energy conservation, i.e., *A* + *R* + *T* = 1. The intensity of light reflection of the samples was measured by using a spectrometer (UV-3600 Shimadzu) with an integrating sphere attachment to measure the diffuse reflectance in the wavelength region of 250–2000 nm. The Fourier transform infrared spectrometer (Spectrum GX PerkinElmer) was used to measure the reflectance spectra in the wavelength region of 2–20 μm. The surface topography of the Cu layer and the 4-layered film samples was measured by AFM (atomic force microscope, XE-100 PARK) within a test area of 5 × 5 μm^2^.

## Additional Information

**How to cite this article:** Hu, E.-T. *et al*. High photon-to-heat conversion efficiency in the wavelength region of 250-1200 nm based on a thermoelectric Bi_2_Te_3_ film structure. *Sci. Rep.* **7**, 44614; doi: 10.1038/srep44614 (2017).

**Publisher's note:** Springer Nature remains neutral with regard to jurisdictional claims in published maps and institutional affiliations.

## Figures and Tables

**Table 1:** Thermal emittance *ε* (0, *T*) reduced by using the measured reflectance spectra under nearly normal incidence condition.

*T*(K)	300	400	500	600
*ε* (0, *T*)	0.013	0.023	0.039	0.064
